# Expanding the evidence base for global recommendations on health systems: strengths and challenges of the OptimizeMNH guidance process

**DOI:** 10.1186/s13012-016-0470-y

**Published:** 2016-07-18

**Authors:** Claire Glenton, Simon Lewin, Ahmet Metin Gülmezoglu

**Affiliations:** 1 Global Health Unit, Knowledge Centre for the Health Services, Norwegian Institute of Public Health, Oslo, Norway; 2 Health Systems Research Unit, South African Medical Research Council, Cape Town, South Africa; 3 Department of Reproductive Health and Research, including the UNDP/UNFPA/UNICEF/WHO/World Bank Special Programme of Research, Development and Research Training in Human Reproduction (HRP), World Health Organization, Geneva, Switzerland

**Keywords:** Health systems, Guidelines, Guidance, Qualitative research, Systematic reviews, GRADE, GRADE-CERQual, Decision-making

## Abstract

**Background:**

In 2012, the World Health Organization (WHO) published recommendations on the use of optimization or “task-shifting” strategies for key, effective maternal and newborn interventions (the OptimizeMNH guidance). When making recommendations about complex health system interventions such as task-shifting, information about the feasibility and acceptability of interventions can be as important as information about their effectiveness. However, these issues are usually not addressed with the same rigour. This paper describes our use of several innovative strategies to broaden the range of evidence used to develop the OptimizeMNH guidance. In this guidance, we systematically included evidence regarding the acceptability and feasibility of relevant task-shifting interventions, primarily using qualitative evidence syntheses and multi-country case study syntheses; we used an approach to assess confidence in findings from qualitative evidence syntheses (the Grading of Recommendations, Assessment, Development and Evaluation-Confidence in Evidence from Reviews of Qualitative Research (GRADE-CERQual) approach); we used a structured evidence-to-decision framework for health systems (the DECIDE framework) to help the guidance panel members move from the different types of evidence to recommendations.

**Results:**

The systematic inclusion of a broader range of evidence, and the use of new guideline development tools, had a number of impacts. Firstly, this broader range of evidence provided relevant information about the feasibility and acceptability of interventions considered in the guidance as well as information about key implementation considerations. However, inclusion of this evidence required more time, resources and skills. Secondly, the GRADE-CERQual approach provided a method for indicating to panel members how much confidence they should place in the findings from the qualitative evidence syntheses and so helped panel members to use this qualitative evidence appropriately. Thirdly, the DECIDE framework gave us a structured format in which we could present a large and complex body of evidence to panel members and end users. The framework also prompted the panel to justify their recommendations, giving end users a record of how these decisions were made.

**Conclusions:**

By expanding the range of evidence assessed in a guideline process, we increase the amount of time and resources required. Nevertheless, the WHO has assessed the outputs of this process to be valuable and is currently repeating the approach used in OptimizeMNH in other guidance processes.

## Background

In 2012, the World Health Organization (WHO) published evidence-based recommendations regarding the use of optimization or “task-shifting” strategies for key, effective maternal and newborn interventions (OptimizeMNH) [1]. Typically, task-shifting involves training lower level health worker cadres to deliver tasks that are normally delivered by higher level health worker cadres. The main aim of this strategy is to increase access to care for people living in areas with a shortage of health workers.

While task-shifting strategies have the potential to increase access to care in settings with critical shortages of specialised health workers, there is also concern that the delivery of health care by health workers with less training could cause harm to people already in vulnerable situations. The effectiveness and safety of such interventions therefore needs to be rigorously evaluated. However, task-shifting strategies that have been assessed as effective and safe in the context of experimental studies may be challenging to implement at scale within a health system. The shifting of tasks from one group of health workers to another can involve social, behavioural and organisational change and, like other complex interventions, its success may depend not only on characteristics of the intervention itself but also on characteristics of the systems in which the intervention is implemented or on interactions between properties of the intervention and systems [2–4]. The success of task-shifting strategies is also likely to be influenced by factors such as the training, supervision and support provided to health workers as well as access to supplies and referral systems. The culture and organisation of healthcare delivery in a particular setting, as well as views and expectations among stakeholders such as service users, the providers involved in task-shifting and managers, may also play important roles in the success of these types of strategies [5–7]. Furthermore, the shifting of tasks can have serious implications for health worker motivation, for instance because of changes in working conditions and salaries, and for health service users’ experience of care [8, 9].

As these issues can represent important challenges to successful implementation, information about an intervention’s feasibility and acceptability can therefore be as important as information regarding intervention effectiveness and safety when recommendations are made. Initiatives that aim to improve the quality of guidelines, such as the AGREE II instrument, also emphasise the importance of seeking stakeholders’ views and preferences, and of discussing potential organisational barriers to the application of recommendations [10]. But despite their potential importance, questions about acceptability and feasibility are usually not addressed with the same rigour as questions about intervention effectiveness. Instead, it appears that these issues are often left to the opinions and experiences of guideline panel members. While this input is useful as part of a dialogue [11], it is limited by the composition of the group. For global guidelines such as OptimizeMNH, the input of panel members from specific geographic or clinical settings and with experience of particular population groups may not give sufficient insight into the values and experiences of service users and other stakeholders more broadly. In addition, the perspectives of some panel members may take precedence over other members because of perceived differences in social status within the guideline panel [12, 13].

As the technical team responsible for developing the OptimizeMNH guidance (Table 1), we saw a need to address these issues in the evidence-to-decision process. In this paper, we describe our use of innovative strategies to expand the evidence used as a basis for WHO recommendations and discuss our experiences with these strategies.

**Table 1:** Defining guidance

Guidance in the context of health systems can be defined as “systematically developed statements produced at global or national levels to assist decisions about appropriate options for addressing a health systems challenge in a range of settings and to assist with the implementation of these options and their monitoring and evaluation” [49].
The term “guidance” can be seen as preferable to the more prescriptive term “guidelines” as health systems, public health and other complex interventions, and the evidence on these issues are often very context sensitive. Health systems guidance statements (which are also sometimes referred to as recommendations) would usually include policy options accompanied by assessments of the quality of evidence supporting them, including evidence of unintended consequences and of acceptability and feasibility issues and resource use. Guidance statements may also be accompanied by discussions of implementation and contextual issues.

Adapted from [49]

## Methods

In developing the OptimizeMNH guidance, we followed the standard procedures set out in the WHO’s Handbook for Guideline Development [14]. These procedures (which include the formulation of questions and outcomes; the retrieval, assessment and synthesis of evidence; the development and formulation of recommendations; and finally, the dissemination and implementation of these recommendations) are intended to ensure that WHO recommendations address key needs and are evidence-based [15]. However, we also elaborated on these standard procedures by adopting innovative approaches that could help us address our goal of producing evidence-informed recommendations that are informed by a broader set of evidence than data on effectiveness alone. These approaches were used at different stages of guidance development: when gathering the evidence to support the guidance, when assessing and synthesising this evidence, and when developing the recommendations (Table 2). The approaches included the following:

**Table 2:** Innovative strategies and resources available to guidance developers

Stage of the guidance	Innovative approaches used in the OptimizeMNH guidance	Areas of use	Resources available to guideline organisations interested in using similar approaches
Gathering the evidence	Systematic reviews of qualitative research (also referred to as qualitative evidence syntheses)	Can be used to: - Gather evidence about stakeholders’ views and preferences, for instance, on which outcomes that stakeholders value the most in relation to the guidance question/s - Gather evidence about the acceptability and feasibility of interventions - Offer information about implementation considerations	- WHO Handbook for guideline development, chapter 15 on using evidence from qualitative research to develop WHO guidelines: http://www.who.int/kms/guidelines_review_committee/en/ - Developing NICE guidelines: the manual: https://www.nice.org.uk/article/pmg20/chapter/1-Introduction-and-overview - Website of the Cochrane Qualitative and Implementation Methods Group, which includes guidance on conducting qualitative evidence syntheses: http://methods.cochrane.org/qi/supplemental-handbook-guidance - Texts on conducting qualitative evidence syntheses: [33, 35]
	Multi-country case study syntheses	Can be used to gather evidence about the issues mentioned above and may be particularly useful where reviews of qualitative research do not cover sufficiently macro-level issues	- Texts on conducting case study syntheses: [22, 42, 50]
Assessing and synthesising the evidence	GRADE-CERQual (“Confidence in Evidence from Reviews of Qualitative Research”) approach	Used to transparently assess and describe how much confidence to place in findings from qualitative evidence syntheses	- Website with information about the GRADE-CERQual approach: www.cerqual.org - Text describing the GRADE-CERQual approach: [26]
Developing the recommendations	DECIDE evidence-to-decision framework	A structured health system framework to help guidance panel members move from evidence to recommendations. For each guidance question, the framework presented, in a structured format, a summary of the evidence regarding: • The benefits and harms of the intervention • Anticipated resource use • Acceptability of the intervention, i.e. the extent to which that intervention is considered to be reasonable among those receiving, delivering or affected by the intervention • Feasibility of the intervention, i.e. the likelihood that the intervention can be properly carried out or implemented in a given context	- Website: https://ietd.epistemonikos.org/#/login - Texts describing the DECIDE framework: [30, 31]

- Broadening the range of evidence used in the guidance by including systematic reviews of qualitative research, multi-country case study syntheses and results from a primary qualitative study
- Using the Grading of Recommendations, Assessment, Development and Evaluation-Confidence in Evidence from Reviews of Qualitative Research (GRADE-CERQual) approach to assess our confidence in the evidence from systematic reviews of qualitative research
- Using a structured health systems framework (the DECIDE framework) to help the guidance panel move from this wider range of evidence to recommendations

## Results

### Gathering the evidence—broadening the range of evidence included in the guidance

The WHO’s Handbook for Guideline Development describes not only several types of questions that can legitimately be addressed in a WHO guideline, including the effectiveness or efficacy of an intervention, but also the negative consequences of the intervention, its social acceptability and its cost-effectiveness. The Handbook describes how qualitative evidence synthesis can be used to explore contextual barriers as well as values and preferences [14]. The main focus of the Handbook is, however, on the use of evidence about effectiveness and, in practice, most WHO guidelines have focused almost exclusively on this question.

For OptimizeMNH, we systematically assessed the effectiveness and safety of the relevant task-shifting interventions through systematic reviews and the use of GRADE. In addition, we used an innovative approach in this stage of guidance development by systematically including other types of evidence. Specifically, we compiled evidence on the acceptability of the interventions among health workers, recipients of care, policy makers and other stakeholders and on the feasibility of these interventions, taking into account the organisational changes to health systems that each task-shifting intervention would entail.

While systematic reviews of randomised trials are seen as the most robust source of evidence when assessing intervention effectiveness, this approach is not well-suited for exploring issues surrounding intervention acceptability and feasibility, particularly for more complex health and social interventions [2, 16]. To answer these questions, we included systematic reviews of qualitative studies (sometimes called qualitative evidence syntheses) as our primary source of evidence [17]. Together with colleagues, we co-authored four qualitative evidence syntheses focusing on factors affecting the implementation of task-shifting among lay health workers, midwives, doctors and nurses [18–21].

The qualitative evidence syntheses provided us with information that was clearly relevant to the questions we had asked. However, most of the studies were of relatively small-scale or pilot programmes, often implemented in the context of research, and focused on factors unfolding at the level of programme delivery in communities and primary care facilities. We decided therefore to complement this evidence with two multi-country case study syntheses focusing on factors affecting the implementation of large-scale programmes. When selecting these programmes, we aimed for broad geographical representation and looked for countries in Africa, Asia and South America that had implemented large-scale programmes. To make the synthesis manageable, we limited ourselves to seven programmes and only selected programmes where a reasonable amount of English-language documentation was available. The multi-country case study syntheses aimed to identify “upstream”, system-level factors associated with programme policies, governance, financing, planning, management and organisation [1, 22]. These case studies synthesised evidence from a variety of sources, including peer-reviewed qualitative and quantitative studies, programme reports, web sites and information from personal communication with individuals familiar with the selected task-shifting programmes.

Another limitation of the evidence we identified through the qualitative evidence syntheses was that it focused primarily on the views and experiences of service users and healthcare providers but paid less attention to the viewpoints of programme managers, policy makers and other stakeholders. We attempted to address this imbalance by carrying out our own primary study. We undertook qualitative analyses of the views and experiences of those contributing to the “Health Information For All By 2015” electronic discussion list, which covers a diverse membership of programme managers, healthcare providers, policy makers, academics and others from 170 countries [23]. These analyses evaluated opinions about how the roles of healthcare providers could be optimised to improve maternal and newborn health in LMICs and the implications of such role optimization [1, 24].

When presenting the evidence from the qualitative evidence syntheses, the country case studies and the primary qualitative studies, we attempted to extract findings that were relevant to specific recommendations being considered by the guidance panel and that concerned specific tasks and groups of health workers. These findings were used to (a) support the guidance panel when reaching recommendations and (b) develop information for end users of the guidance regarding implementation considerations for each recommendation. Tables 3 and 4 provide examples of how these findings informed the development of recommendations in the OptimizeMNH guidance.

**Table 3:** Example of how the qualitative evidence informed the final recommendations in the OptimizeMNH guidance (1): provision of continuous support during labour by lay health workers, in the presence of a skilled birth attendant

The guidance panel was asked to consider whether lay health workers could provide continuous support, such as emotional and practical support, during labour, while in the presence of a skilled birth attendant providing the necessary clinical care.
Information regarding benefits and harms came from a systematic review of trials [51]. This evidence suggested that this support, when provided by lay health workers or other birth supporters, may have important health benefits (low to moderate certainty evidence). Based on discussion in the technical team, we also concluded that the intervention would require little additional training, supervision and supplies.
Information regarding acceptability and feasibility came from two syntheses of qualitative evidence [19, 20]. The evidence suggested that mothers appreciated this support from lay health workers and that health professionals working alongside lay health workers often appreciated their contribution to their busy workload and their skills in communicating with mothers (moderate confidence in the evidence). However, the relationships between lay health workers or other birth supporters and professional midwives was sometimes ambivalent and, at times, conflictual, possibly because the midwife role was shifted in a more medical direction (moderate confidence in the evidence). Having to be present during labour and birth could also lead to irregular and unpredictable working conditions for the lay health worker, which might have implications for their expectations regarding incentives (low confidence in the evidence) and could lead to concerns about personal safety when working in the community or travelling at night (low confidence in the evidence).
This information was presented to the guidance panel in a summarised form using the DECIDE evidence to decision framework. More detailed versions were presented in appendices using summary of findings tables and full versions of each review were also made available to the panel.
Based on this evidence, the panel decided to recommend the intervention. Potential challenges regarding the acceptability of the intervention to lay health workers and other healthcare providers were highlighted under “Implementation considerations”.

Adapted from [1]

**Table 4:** Example of how the qualitative evidence informed the final recommendations in the OptimizeMNH guidance (2): provision of vasectomy by trained midwives

The guidance panel was asked to consider whether midwives could perform vasectomies.
We were unable to identify any eligible studies that assessed the benefits or harms of midwives performing vasectomies. We did have indirect evidence from one systematic review of trials [52] that there may be little or no difference between midwives and doctors with regard to complications during surgery or postoperative morbidity for tubal ligation (low quality evidence).
Based on discussion in the technical team, we concluded that the intervention would require additional training, supervision and supplies and a functioning referral system for failed vasectomies or complications and might also require changes to norms and regulations.
Information regarding acceptability and feasibility came from one synthesis of qualitative evidence [20]. The synthesis did not identify any studies that evaluated the acceptability of vasectomy when performed by midwives. For other midwife-delivered interventions, the synthesis suggested that midwives and their trainers generally felt that midwives had no problem learning new clinical techniques (moderate confidence in the evidence) and might be motivated by being “upskilled” as this could lead to increased status, promotion opportunities and increased job satisfaction (moderate confidence in the evidence). However, midwives might be unwilling to take on tasks that moved beyond obstetric care, such as tasks related to family planning, possibly because this was not viewed as part of their role and might entail an increased workload (moderate confidence in the evidence). In addition, a lack of clarity in roles and responsibilities between midwives and other healthcare providers, as well as status and power differences, might also lead to poor working relationships and “turf battles” (moderate confidence in the evidence). Finally, the synthesis suggested that additional training and supervision were often insufficient in midwife task-shifting programmes.
This information was presented to the guidance panel in a summarised form using the DECIDE framework. More detailed versions were presented in appendices using summary of findings tables and full versions of each review were also made available to the panel.
Based on this evidence, the panel decided to recommend the intervention only in the context of rigorous research. The panel further specified that implementation in the context of research should only be done where a well-functioning midwife programme already exists and a well-functioning referral system is in place or can be put in place.

Adapted from [1]

We also wanted to offer those using the OptimizeMNH guidance more general information about the acceptability and feasibility of task-shifting strategies. We therefore carried out a “cross-cutting” analysis of findings from all of the sources described above. This cross-cutting analysis provided evidence regarding task-shifting in general where no direct evidence was available for specific task-shifting interventions. The cross-cutting analysis also contributed to a chapter in the WHO OptimizeMNH guidance on overarching implementation considerations.

### Assessing and synthesising the evidence—introducing an approach to assess our confidence in findings from qualitative evidence syntheses

The WHO Guideline Handbook notes that guideline technical teams need to provide an assessment of the quality of the evidence included in a WHO guideline [14]. For evidence of effectiveness, the WHO uses the GRADE approach to assess quality [25]. GRADE is not, however, designed to be applied to qualitative research, and we were not aware of other systems for assessing quality or confidence in findings from qualitative evidence syntheses or for indicating these assessments to end users. Having made the decision to include qualitative evidence syntheses, we therefore needed an approach in transparently assessing and describing how much confidence to place in findings from these types of syntheses.

Work to develop this approach was carried out in collaboration with other qualitative researchers [26], informed by the principles of qualitative research. We were also influenced by the processes used by the GRADE Working Group, which has given considerable thought to how to assess confidence in evidence from systematic reviews. This work resulted in an approach which we named GRADE-CERQual [26]. Since its initial use in the OptimizeMNH guidance, CERQual is being developed further as part of the GRADE Working Group and is now being used in several other WHO guidance development processes [27].

### Developing the recommendations: using a structured health systems framework

When assessing the different factors that influence recommendations, including effectiveness, acceptability, feasibility, and cost, the Guideline Handbook [14] suggests that the guideline panel makes use of evidence-to-recommendation tables. These tables can be used to lay out what we know about these different factors and can also be used to record the guideline panel’s judgements about these factors and how they contributed to the development of the recommendation [14]. For OptimizeMNH, we piloted an early paper version of a decision table developed by the DECIDE project [28] and the Grade Working Group [29]: the DECIDE Evidence to Decision framework [30, 31].

The DECIDE framework aims to help guidance panel members move from evidence to health system recommendations by informing judgements about the balance of consequences of each option. The framework is based on a review of relevant literature, brainstorming, feedback from stakeholders [32], and the application of the framework to examples.

For each guidance question, the framework presented, in a structured format, a summary of the evidence regarding the following considerations:

- The benefits and harms of the intervention/s (sometimes referred to as “effectiveness and safety”)
- Anticipated resource use
- Acceptability of the intervention/s
- Feasibility of the intervention/s

For each of these elements, the framework also included an assessment of the certainty of the evidence, using GRADE (for evidence of effectiveness) and CERQual (for evidence on acceptability and feasibility from qualitative evidence syntheses). The following were also included for each guidance question:

- A judgement regarding the balance of desirable and undesirable consequences for the intervention/s
- A recommendation and a justification for this
- Implementation considerations for the intervention/s
- Relevant monitoring and evaluation/research priorities in relation to the intervention/s

In addition to the evidence collated in the DECIDE frameworks, full evidence profiles for the reviews of the effectiveness of interventions, as well as summaries of findings for the qualitative evidence syntheses on the acceptability, feasibility and implementation of these interventions, were made available to the guidance panel. These summaries were, in turn, linked to full systematic reviews. The DECIDE Team also prepared a video to help guidance panel members understand the purpose of the framework and how to use it. This was made available prior to the guidance panel meeting and also shown at the start of the meeting.

## Discussion

### Broadening the range of evidence included in guidance: lessons learnt

The first innovative approach that we used in the development of the OptimizeMNH guidance was the systematic inclusion of additional types of evidence to complement the evidence of effectiveness (Table 5). This approach had a number of implications. On a practical level, broadening the scope of evidence to be considered in relation to each guidance question required more time and resources. It also required a wider set of synthesis, assessment and interpretation skills within the technical team preparing the documents for the guidance panel. In our case, these skills were represented within the technical team. Other technical teams using a similar guidance development approach may not have the skills to undertake qualitative evidence syntheses or country case study syntheses but will at least need skills in commissioning and critically appraising such evidence. Creating technical teams with these skills may be challenging as there are few groups with extensive experience in implementing them. However, the number of researchers with the appropriate skills is increasing rapidly, and guidance is now well-developed [33–35].

**Table 5:** Overview of the different types of evidence used in the OptimizeMNH guidance development process

What type of evidence did we use?	Which part of the DECIDE evidence-to-decision framework was it used to address?	What type of evidence did it consider?
Systematic reviews of effectiveness	- What are the benefits and harms of the different task-shifting options?	The reviews primarily included randomised trials of task-shifting interventions. In some reviews, non-randomised study designs were also included.
Qualitative evidence syntheses	- Is the task-shifting option acceptable to most stakeholders? - Is the task-shifting option feasible to implement?	The qualitative evidence syntheses included primary studies of task-shifting that had used qualitative methods for data collection and for data analysis
Multi-country case study syntheses	- Is the task-shifting option acceptable to most stakeholders? - Is the task-shifting option feasible to implement?	The multi-country case study syntheses reviewed evaluations and studies of large-scale programmes designed to optimise the health workforce in LMICs. Evaluation reports, programme guidelines and published studies were gathered for each selected country programme
Primary research	- Is the task-shifting option acceptable to most stakeholders? - Is the task-shifting option feasible to implement?	The primary research involved qualitative thematic analysis of messages submitted to two email discussion forums. The forums focus on the healthcare information needs of frontline health workers and citizens in LMICs and how these needs can be met and also include discussion of diverse aspects of health systems.

We had originally planned to gather evidence about stakeholder acceptability and feasibility through qualitative evidence syntheses only. As described above, these syntheses, which only included published qualitative studies, primarily offered evidence surrounding factors and stakeholders at the community or primary healthcare facility level. This led us to carry out the multi-country case studies, where we included a broader set of information sources, and also led us to carry out our own primary study among stakeholders poorly represented in the qualitative evidence syntheses. In retrospect, we think that the findings of this primary study should have been included in the qualitative evidence syntheses rather than being treated as a stand-alone product.

Although this innovation expanded the preparatory work needed for the guidance, we would argue that the inclusion of evidence from qualitative evidence syntheses and country case study syntheses offered relevant information for the guidance panel on the feasibility and acceptability of the interventions. When such evidence is not systematically assessed and included, acceptability and feasibility issues may either be overlooked altogether or incorporated into guideline decisions based only on anecdotal evidence. Our experience from the development of the OptimizeMNH guidance suggests that the inclusion of evidence from qualitative evidence syntheses and country case study syntheses reduced the use of anecdotal evidence by guidance panel members when assessing the balance of consequences for each guidance question. In addition, our use of such evidence appeared to address guideline panel members’ perceptions that the WHO often over-emphasises evidence from randomised trials at the expense of evidence from programme experience. One additional benefit of synthesising relevant qualitative evidence was that we were able to use this to develop implementation considerations for task-shifting in maternal and newborn health [1].

### Introducing an approach to assess confidence in findings from qualitative evidence syntheses: lessons learnt

Our second innovation was the use of the GRADE-CERQual approach in order to systematically and transparently assess our confidence in findings from the qualitative evidence syntheses conducted to inform the guidance.

Our use of CERQual was received well by the guidance panel, in part because it addressed the need to systematically incorporate a wider range of evidence into the guidance process. The approach also had a number of advantages. When making recommendations, all guidance panels need to take into account how confident they can be in the underlying evidence. Had we not provided the OptimizeMNH panels with the CERQual assessments of our confidence in the findings from the qualitative evidence syntheses, they would have had to make their own judgments and may not have done this in a systematic way. The use of CERQual also allowed us to identify and highlight gaps in the evidence. For instance, where we assessed the confidence in the evidence for a qualitative finding to be low or very low, this was an indication that further research was needed in this area. These research gaps were also reflected in the information presented to the guidance panel and carried through to the recommendations.

We were initially concerned that using the CERQual approach would confuse guidance panel members as they would not have encountered this approach before. In practice, this did not appear to be a problem, probably because members were already familiar with GRADE for assessing findings regarding the effectiveness of interventions, which uses the same principles, and because the panel was briefed on the approach at the start of the meetings.

Some of the findings contributing to OptimizeMNH were based on a very wide range of evidence types. In particular, the country case studies included both qualitative and quantitative studies as well as programme reports, which often did not provide detailed descriptions of methods used. Using CERQual to assess our confidence in the findings from the country case study syntheses was not feasible as the CERQual approach is not designed, at present, to accommodate this range of evidence [26].

### Using a structured health systems framework: lessons learnt

Our final innovation was the use of DECIDE evidence-to-decision frameworks [36] to present evidence to the guidance panel. There were several advantages in using this structured framework. Firstly, the framework helped panel members to think through each of the considerations that might be important in making a recommendation and ensured that key considerations were taken into account. The concise format also appeared to help focus and structure the discussions and may have improved the use of the limited time that the guidance panel had to weigh the balance of consequences and make judgements about complex questions regarding task-shifting. Because the framework prompted the guidance panel to justify all of the recommendations made, we believe that the transparency of the decision-making process was improved. Furthermore, end users of the guidance have access to a clear record of recommendation decisions and how these were made. The framework also contributes to closing the knowledge-to-action cycle by capturing implementation considerations and highlighting evidence gaps where more research is needed [37, 38].

The use of the evidence-to-decision frameworks also led to a number of challenges. First of all, preparation of the frameworks took considerable time in advance of the panel meeting, although this may have saved time later on when preparing the final guidance document. Secondly, because the DECIDE framework is a new tool, it required some explanation in advance of the panel meetings. However, panel members appeared to grasp the function and content quickly, and the informal feedback on the framework was positive.

Because the OptimizeMNH guidance included a very large number of recommendations (128 in total), the amount of time available in the panel meetings to discuss each evidence-to-decision framework was limited. The inclusion of additional types of evidence also added to the amount of information that panel members were expected to read. Nonetheless, our experience suggests that the structured format of the DECIDE framework allowed the technical team to present a large and complex body of evidence to panel members in a fairly straightforward and easy-to-assimilate way. Further work is now needed to explore guidance panels’ views of such evidence-to-decision frameworks and to examine how the presentation of evidence in these formats impacts on the deliberations and decisions of guidance panels and on the transparency to users of these decisions.

## Conclusion

In this paper, we have described our use of a number of innovative strategies to expand the range of evidence used to develop WHO global recommendations and discussed our experiences with these strategies. Expanding the range of evidence contributing to a guidance process increases the amount of time and resources needed and the range of skills required within the technical team developing the guidance. However, our experience with the OptimizeMNH guidance suggests that such efforts may be valuable, particularly for recommendations on more complex health and social interventions. This view is supported by efforts within WHO to replicate the OptimizeMNH approach in other guidance processes [27]. When considering whether to adopt a similar approach, producers of guidance should assess the extent to which acceptability and feasibility issues are likely to be important enough to influence their recommendations. So far, the WHO is using this approach in recommendations in guidance on more complex health systems and behavioural interventions, but acceptability and feasibility issues may be equally relevant for clinical interventions. In fact, we would suggest that there are few situations where these issues are not likely to be relevant, and even in situations where guidance developers feel confident that they already have a sufficient overview of these issues and additional evidence is not required, a framework approach where this is made transparent can still be useful. We have developed a list of questions (Table 6) to help groups involved in developing guidance decide whether additional evidence syntheses are needed. Further research is needed to refine these questions and to develop an understanding of the typical time and resources required to expand the range of evidence sources used to develop guidance. Table 2 also provides links to resources available to guidance developers who are considering expanding the range of syntheses that will be used to inform the guidance.

**Table 6:** When to consider expanding the range of evidence syntheses undertaken to inform the development of a guideline or guidance

Principles:
• For most interventions for which guidance is developed, including clinical, health systems and public health interventions, guidance panels should consider how different stakeholders value different outcomes; the effectiveness, acceptability and feasibility of the intervention; implications for resource use; equity impacts; and implementation considerations • A structured evidence-to-decision framework, such as the DECIDE framework [30, 31], should be used to guide these considerations, even if it is likely that evidence syntheses will not be needed for some of the considerations included in these frameworks (see below)
When to consider expanding the range of evidence syntheses informing guidance development:
• Expanding the range of evidence syntheses beyond evidence of effectiveness should be considered under the following circumstances: ◦ If the guidance is considering a new intervention, an intervention for which guidance has not previously been developed, or an intervention for which the implementation mechanism/s have changed ◦ If there are reasons to anticipate that feasibility, acceptability, resource use and/or equity are likely to be important considerations for the interventions included in this guidance. These considerations may be particularly important for interventions directed at health systems or other systems; interventions that focus on changing people’s views or behaviours; and interventions that have multiple components and long causal pathways or involve multiple actors or systems. Note that stakeholders should be involved in discussions on this issue • If a decision has been made that the range of evidence syntheses needed to support the development of guidance should be expanded, consider the following: ◦ Do evidence syntheses already exist that could be used to address the considerations identified? ◦ Will existing evidence syntheses require additional work, for example, to assess how much confidence to place in the synthesis findings and/or to undertake specific subgroup analyses? ◦ If existing evidence syntheses are not available for some or all of the considerations to be explored by the guidance: ▪ Do the timeline and resources available for the guidance development allow for new syntheses to be undertaken? ▪ If so, which are the highest priority considerations for new syntheses and what should be the scope of these syntheses? ▪ What skills are likely to be required to undertake these syntheses? Are the skills to undertake these syntheses available within the lead organisation for the guidance or do they need to be commissioned externally? ▪ What financial and other resources are needed to conduct these syntheses within the timeframe for development of the guidance? ▪ Could the process of conducting multiple evidence syntheses be made more efficient by combining database searching and screening for several of the syntheses, such as those syntheses focusing on intervention effectiveness, acceptability, feasibility and resource use considerations? • Once the evidence syntheses are underway or available, consider the following: ◦ What tools are needed to assess how much confidence to place in the synthesis findings (for instance, the GRADE approach)? Will these assessments be conducted by the review teams, by the guidance commissioners or by a third party? Who will be responsible for summarising the available evidence in order to populate an evidence-to-decision framework for each guidance question?

As more qualitative evidence syntheses are produced, methods for synthesising this type of evidence are becoming more sophisticated and the number of people able to produce these types of syntheses is growing. These developments will make the process of including qualitative evidence in evidence-to-decision processes easier in the future. However, a number of challenges remain, as discussed above. Elsewhere, we have described how evidence from qualitative research can be used in developing guidelines [39], including to help shape a guideline’s key questions by informing the populations, interventions, comparators and outcomes on which each key question should focus and to understand how different stakeholders value different outcomes. We did not use these additional strategies in OptimizeMNH, but they have been part of the development for a forthcoming WHO guideline on antenatal care [40].

Our experience from OptimizeMNH suggests that relevant programmatic experience regarding “upstream” systems-level factors is often unexplored or undocumented using traditional research approaches. Researchers should explore these issues to a greater extent [22]. Alongside this, we need to develop better methods for identifying and synthesising information from programme reports and other grey literature as well as methods for assessing our confidence in syntheses based on this type of data [41, 42].

The OptimizeMNH experience suggests that the DECIDE evidence-to-decision frameworks are a useful way of guiding panels through a wide range of evidence and towards the development of transparent recommendations. We have learnt a great deal about how to populate these frameworks with evidence of the effectiveness, feasibility and acceptability of health interventions. However, more research is needed, in particular on how these kinds of evidence are used by guidance panels in their decision-making and how different kinds of evidence influence the final recommendations made. In addition, we need further worked examples of approaches for including evidence regarding resource use and cost-effectiveness. This is one area that those involved in the DECIDE work are exploring [36].

Finally, we need to explore how this type of health system guidance can best be disseminated to and utilised by end users, including national and regional policy makers and programme planners. For most health systems questions, evidence regarding effectiveness, acceptability, feasibility, and resource use is all likely to be context-specific to some degree. End users therefore need help in adapting the recommendations to their own contexts. The OptimizeMNH guidance includes a workbook that specifically aims to help end users contextualise the recommendations [43]. However, more work is needed on tools to contextualise global and national research and guidance for implementation in specific settings [44–47] and on ways of appraising such guidance [48].
